# South African Society of Clinical Microbiology *Clostridioides difficile* infection diagnosis, management and infection prevention and control guideline

**DOI:** 10.4102/sajid.v35i1.219

**Published:** 2020-10-28

**Authors:** Trusha Nana, Chanelle Moore, Tom Boyles, Adrian J. Brink, Joy Cleghorn, Lesley M. Devenish, Briette du Toit, Ernst S. Fredericks, Molebogeng R. Lekalakala-Mokaba, Caroline Maluleka, Muhammad N. Rajabally, Gary Reubenson, Liliwe Shuping, Karin Swart, Khine Swe Swe Han, Jeannette Wadula, Justyna Wojno, Warren Lowman

**Affiliations:** 1 Department of Clinical Microbiology and Infectious Diseases, Faculty of Health Sciences, University of the Witwatersrand, Johannesburg, South Africa; 2 Charlotte Maxeke Johannesburg Academic Hospital Microbiology Laboratory, National Health Laboratory Services, Johannesburg, South Africa; 3 Ampath Laboratories, Johannesburg, South Africa; 4 Department of Medicine, Faculty of Health Sciences, University of the Witwatersrand, Johannesburg, South Africa; 5 Department of Infectious and Tropical Diseases, London School of Hygiene and Tropical Medicine, London, United Kingdom; 6 Department of Medical Microbiology, Faculty of Health Sciences, University of Cape Town, Cape Town, South Africa; 7 Life Healthcare Group, Johannesburg, South Africa; 8 Infection Control Services Laboratory, National Health Laboratory Services, Johannesburg, South Africa; 9 Mediclinic Southern Africa, Cape Town, South Africa; 10 Department of Physiology, Faculty of Science, Nelson Mandela University, Port Elizabeth, South Africa; 11 Department of Microbiology, Faculty of Health Sciences, Sefako Makgatho Health Sciences University, Pretoria, South Africa; 12 Dr George Mukhari Academic Hospital Microbiology Laboratory, National Health Laboratory Services, Pretoria, South Africa; 13 Mediclinic Constantiaberg, Cape Town, South Africa; 14 Department of Paediatrics and Child Health, Faculty of Health Sciences, University of the Witwatersrand, Johannesburg, South Africa; 15 Centre for Healthcare-Associated Infections, National Institute for Communicable Diseases, a division of National Health Laboratory Service, Johannesburg, South Africa; 16 Netcare Hospitals Limited, Johannesburg, South Africa; 17 Medical Microbiology Department, Inkosi Albert Luthuli Central Hospital Academic Complex, National Health Laboratory Services, Durban, South Africa; 18 Department of Medical Microbiology, School of Laboratory Medicine and Medical Sciences, University of KwaZulu-Natal, Durban, South Africa; 19 Chris Hani Baragwanath Hospital Microbiology Laboratory, National Health Laboratory Services, Johannesburg, South Africa; 20 Lancet Laboratories, Cape Town, South Africa; 21 Department of Clinical Microbiology, PathCare/Vermaak Pathologists, Johannesburg, South Africa; 22 Department of Clinical Microbiology and Infection Prevention and Control, WITS Donald Gordon Medical Centre, Johannesburg, South Africa

**Keywords:** *Clostridioides difficile*, diagnosis, treatment, surveillance, infection control, infection prevention, outbreak

## Abstract

*Clostridioides difficile* infection (CDI) is a problem in both developed and developing countries and is a common hospital-acquired infection. This guideline provides evidence-based practical recommendations for South Africa and other developing countries. The scope of the guideline includes CDI diagnostic approaches; adult, paediatric and special populations treatment options; and surveillance and infection prevention and control recommendations.

## Introduction

*Clostridioides difficile* infection (CDI) is a global problem, with antibiotic exposure being one of its key drivers.^1,2,3^ Distinguishing colonisation from disease is of paramount importance to avoid unnecessary therapy. Severe CDI is associated with significant morbidity and mortality.^4^ In addition, the management of recurrent disease can be challenging.^5^ New CDI treatment options have become available. Evidence regarding the efficacy of the various treatment options has resulted in recent changes to recommended treatment. *Clostridioides difficile* infection surveillance, particularly for healthcare-onset (HO) CDI, using standardised definitions is a priority. Implementation of appropriate infection prevention and control (IPC) measures is essential to minimise transmission.

The 2017 Infectious Diseases Society of America (IDSA) and the Society for Healthcare Epidemiology of America (SHEA) CDI guidelines provide a comprehensive overview.^6^ This South African guideline incorporates updated information and recommendations based on local context and resources. The guideline aims to provide pathologists, clinicians and IPC practitioners with practical, evidence-based advice.

## Methodology

A committee comprising members from South African Society of Clinical Microbiology (SASCM), Infectious Diseases Society of South Africa (IDSSA), Southern African Society of Paediatric Infectious Diaeases (SASPID), South African Antimicrobial Stewardship Programme (SAASP), Infection Control Society of Southern Africa (ICSSA), National Institute of Communicable Diseases (NICD) and South African Gastroenterology Society (SAGES) was convened. The scope of the guideline was discussed through email correspondence and individuals were assigned specific sections. Literature searches were conducted and a meeting to discuss recommendations was held on 01 June 2018. Sections were then collated, edited and revised by T.N., C. Moore, W.L., with subsequent review by the entire committee.

Grading of evidence and the strength of recommendations was based on modification of the grading system used in the South African guideline for the management of community-acquired pneumonia.^7^

### Strength of recommendation

Strong: strong recommendation for or against use.

Weak: weak recommendation for or against use.

### Quality of evidence

High: evidence from at least one randomised controlled trial (RCT), meta-analysis or systematic review.

Moderate: evidence from at least one well-designed clinical trial without randomisation, from cohort or case-control studies, from multiple time series or well-designed diagnostic accuracy studies.

Low: evidence from opinions of respected authorities or based on clinical experience.

## Epidemiology

*Clostridium difficile* has recently been renamed *Clostridioides difficile. Clostridioides difficile* is associated with a spectrum of diseases ranging from asymptomatic colonisation to fulminant infection. It is the most common infectious cause of antibiotic-associated diarrhoea (AAD).^3^ Its infection accounts for 20% – 30% of all cases of AAD. It is a common hospital-acquired infection (HAI).

### Incidence

When interpreting reported CDI rates, the sampling and testing methodology must be considered. A lack of standardised reporting limits comparison of rates between studies. Large-scale, multicentre, multi-country studies are required to provide global epidemiological data.

*Clostridioides difficile* infection epidemiology data from low- and middle-human development index countries including South Africa are limited.^2^ A systematic review of CDI in these regions showed a 15.8% CDI incidence rate amongst symptomatic patients. It appears that the frequency of CDI is lower in these regions than in more developed countries. In a 2013 publication, Groote Schuur Hospital, Cape Town, reported 8.7 cases per 10 000 hospitalisations (hospital-acquired CDI [HA-CDI]).^8^ Hospital-acquired CDI comprised 68% of CDI cases at this institution, with the remainder being community-acquired CDI (CA-CDI). Antibiotic use was identified as the main risk factor for HA-CDI and inflammatory bowel disease (IBD) for CA-CDI. Two cases of the Polymerase Chain Reaction (PCR) ribotype 027 (North American pulsed field type 1 [NAP1] or restriction endonuclease group BI analysis) were identified. At Charlotte Maxeke Johannesburg Academic Hospital, over an 18-month period during 2013–2014, 154 CDI cases were identified from the gastroenterology, infectious diseases and intensive care units.^9^ The majority of cases were HA-CDI, with CA-CDI representing only 1.3%. Exposure to antibiotics in the previous 30 days was the most common risk factor, which was present in 97.9% of cases.

A large multicentre European study reported an overall CDI incidence of 7/10 000 patient days for 2012–2013. However, there were substantial inter-country differences (0.7–28.7/10 000 patient days).^10^ There were many different ribotypes, with 027 being the most common (18.5% of tested isolates). The LuCID study from 2014 to 2015, which included France, Italy and the United Kingdom (UK) (59 hospitals), found a mean annual CDI rate of 2.5/10 000 patient days.^11^ The United States Centers for Disease Control and Prevention (CDC) Emerging Infections Programme reported a CDI incidence of 142.61/100 000 persons for 2016.^12^ *Clostridioides difficile* infection was the most common HAI in the United States of America (USA) in 2011.^13^ A meta-analysis of CDI incidence in Asia found significant regional differences, with higher rates in East Asia.^14^ The pooled incidence from 11 studies included in the analysis was 5.3/10 000 patient days. The proportion of infections caused by the 027 ribotype was low (0.0% – 2.1%).

Emergence of the 027 ribotype strain in early 2000s was associated with increased incidence and severity of the disease in Canada, the USA, parts of Europe and Asia.^1,15^ As a result of the increased morbidity and mortality attributed to infection with this ribotype, it is considered a hypervirulent strain. Ribotype 027 has been linked to increased transmissibility and has been associated with numerous outbreaks. Its fluoroquinolone resistance coupled with the widespread usage of fluoroquinolones has likely also contributed to its spread. The prevalence of this ribotype in the UK had decreased to 2.3% by 2012–2013.^10^ Ribotype 078 is another hypervirulent strain. A 2008 study from the Netherlands described an increase in the prevalence of this ribotype and CDI disease severity similar to 027 infection.^16^ Ribotype 078 has been found in various parts of Europe and the USA.^1,17^

### Community-acquired *Clostridioides difficile* infection

The reported incidence of CA-CDI has increased significantly in the last decade.^18^ In some regions, CA-CDI accounts for up to 41% of all CDI cases.^18^ Community-acquired CDI is frequently seen in younger patients without co-morbidities and in one-third of patients with CA-CDI antibiotic exposure is absent. It may be associated with outpatient healthcare visits, antibiotic use and IBD.

### Morbidity and mortality

*Clostridioides difficile* infection is associated with a significant impact on quality of life, morbidity, healthcare utilisation and mortality, particularly in older patients, those with severe disease, those infected with hypervirulent strains and recurrent CDI (rCDI) episodes.^4^

### Risk factors

The risk for CDI is related to disruption of the gut microbiome, host factors and exposure to *C. difficile.*^10^ Advanced age (which may be a marker of co-morbidities), hospitalisation and antibiotic exposure are the principal risk factors of CDI.^3,4^ Specific patient populations with an increased risk of CDI include haematology-oncology, solid organ transplant, HIV-positive and IBD patients.^19,20^ Other reported risk factors include enteral feeds and gastrointestinal surgery.^21^

Patients who have experienced an initial episode of CDI are at increased risk of subsequent CDI episodes. The risk of recurrence increases further with each successive CDI episode.^5^

## Recommendations

See Table 1 to guide navigation to relevent sections.

**TABLE 1:** Quick reference for recommendations.

Section	Questions	Recommendations
A	1–6	CDI diagnosis
B	7–11	Treatment of initial episode of CDI in adults
C	12–14	Treatment of recurrent CDI in adults
D	15–17	The role of FMT in the treatment of CDI in adults
E	18–19	Treatment of CDI in special risk populations, including IBD
F	20–26	Treatment of CDI in the paediatric population
G	27–29	Surveillance of CDI
H	30–35	CDI prevention and control (IPC)
I	36	Managing CDI in an outbreak setting
J	37–38	Antimicrobial stewardship and CDI
K	39–40	CDI prevention

CDI, *Clostridioides difficile* infection; FMT, faecal microbiota transplantation; IBD, inflammatory bowel disease; IPC, infection prevention and control.

### A. *Clostridioides difficile* infection diagnosis

#### Summary of recommendations

1. **How should the pretest probability of *Clostridioides difficile* infection be calculated?**

No reliable evidence-based method for determining pretest probability is currently available.

2. **Which patients should be tested for *Clostridioides difficile* infection?**

**Recommendation:** Only patients fulfilling the case definition of suspected CDI should be tested (*weak recommendation, low quality of evidence*).

3. **What is the appropriate testing strategy for patients fitting the case definition of suspected *Clostridioides difficile* infection?**

**Recommendation:** Algorithm-based testing is required to optimise both the negative predictive value (NPV) and positive predictive value (PPV) of laboratory results (*strong recommendation, low quality of evidence*).

##### Recommended approaches

**FIGURE 1:**
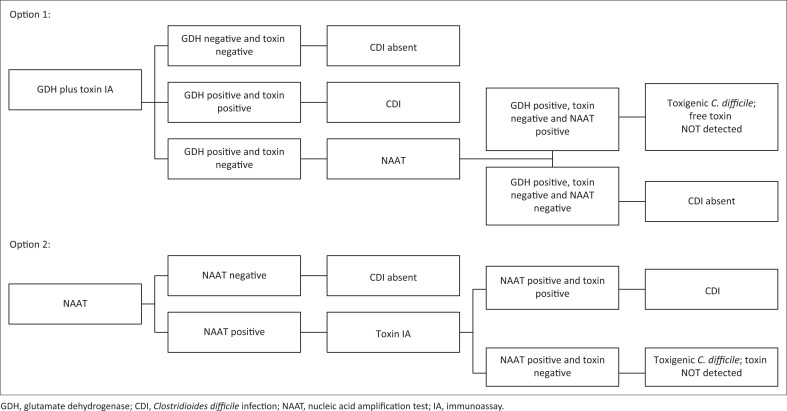
Testing algorithms and results reporting.

1. Glutamate dehydrogenase (GDH) and toxin A/B immunoassay (IA) followed by nucleic acid amplification test (NAAT) for GDH-positive toxin-negative samples OR
2. Nucleic acid amplification test followed by toxin A/B IA for NAAT-positive samples (Figure 1).

4. **How should disease severity be graded?**

##### Recommendation

###### Mild-to-moderate disease

*Clostridioides difficile* infection without features meeting the definition of severe disease.

###### Severe disease

*Clostridioides difficile* infection with white blood cell count of ≥ 15 × 10^9^/L and/or creatinine of > 133 *µ*mol/L but not meeting the criteria for fulminant disease.

###### Complicated/Fulminant

*Clostridioides difficile* infection with the presence of hypotension or ileus or megacolon (*strong recommendation, low quality of evidence*).

5. **What is the definition of recurrent *Clostridioides difficile* infection?**

**Recommendation:** New CDI episode after completion of therapy and within 2–8 weeks following the initial onset of symptoms.

6. **What is the role of repeat testing?**

**Recommendation:** After an initial negative result, repeat testing within 7 days of an initial negative test is not recommended; for suspected recurrence, testing for the presence of free toxin is important; for test of cure, repeat testing is not indicated (*strong recommendation, moderate quality of evidence*).

#### Rationale for recommendations

1. **How should the pretest probability of *Clostridioides difficile* infection be calculated?**

Calculation of the pretest probability of CDI would aid decision-making with respect to the appropriate use of specific tests. Our literature search did not find any studies that had developed and validated a score for calculating the pretest probability of CDI. Development of risk prediction models for CDI should be a research priority.

2. **Which patients should be tested for *Clostridioides difficile* infection?**

Multiple systematic reviews and meta-analyses have described factors that are associated with CDI; however, most of the studies have been conducted in well-resourced settings.^21^ Risk factors can be divided into pharmacological, host and clinical interventions or characteristics, and a non-exhaustive list is presented in Box 1. The most important risk factor for CDI is antibiotic use.^22^ Ampicillin, amoxicillin, cephalosporins, clindamycin and fluoroquinolones are the antibiotics that are most frequently associated with CDI, but almost all antibiotics have been implicated.^23^ The influence of stomach acid suppression remains uncertain; some reports have suggested an increased risk of infection, whereas others, after adjusting for co-existing conditions, have not confirmed such a risk.^24,25^ Other important risk factors are advanced age, IBD, organ transplantation, chemotherapy, chronic kidney disease and immunodeficiency.^21^

BOX 1 Non-exhaustive list of factors associated with *Clostridioides difficile* infection.

**Table:** 

**Pharmacological:** Any use of antibiotics (broad and specific) Use of any antiulcer medication (conflicting evidence) Aspirin Corticosteroids
**Host-related:** Advanced age Chronic kidney disease Immunodeficiency Diabetes mellitus Lymphoma or leukaemia Solid cancer or malignancy Organ transplantation Severity of comorbidity Inflammatory bowel disease Congestive heart disease Chronic obstructive pulmonary disease Peptic ulcer Diverticular disease Gastroesophageal reflux disease Chronic obstructive pulmonary disease Low mean concentration of 25 hydroxyvitamin D
**Clinical interventions or characteristics:** Duration of hospitalisation Nasogastric tube feeding Stay in intensive treatment unit Non-surgical GI procedure

GI, Gastrointestinal.

There is no consensus on the case definition for suspected CDI. The definition presented in Box 2 is based on expert opinion, taking into account the strength of association of risk factors. All patients meeting the case definition should have stool tested for CDI. It is very unlikely that patients not meeting the case definition will have CDI and alternative causes for diarrhoea should be considered.

BOX 2 Case definition of suspected *Clostridioides difficile* infection for patients > 2 years old.

**Table:** 

Inpatients developing new onset diarrhoea† > 48 h after admission to hospital OR
Inpatients who presented with diarrhoea† who continued for 3 days post-admission and, where indicated, appropriate stool testing has not detected a pathogen and there is no other likely cause, for example, laxative use OR
Patients presenting to care with diarrhoea† and ANY of the following: Overnight stay at a healthcare facility within 12 weeks Antibiotics within 12 weeks‡ Inflammatory bowel disease Severe intestinal motility disorders (including Hirschsprung disease) Solid organ transplant Known current malignancy Fitting the definition of fulminant disease

† , The definition of diarrhoea is ≥ 3 stools that take the shape of their container in a 24-h period;

‡ , Refer to text for considerations in children ≤ 2 years.

The case definition applies to adults and most children with some exceptions. In neonates and infants (children less than 1 year of age), colonisation is common and true CDI is extremely rare, so positive tests almost always reflect colonisation and testing is therefore strongly discouraged. Colonisation rates decline with age but remain high in children 1–2 years old. These children should only be tested for CDI once more common causes have been excluded. Unlike in adults, CDI testing is not currently indicated in children with community-associated diarrhoeal disease following recent antibiotic use. *Clostridioides difficile* infection has been described as a community-acquired infection in children; however, most CDI cases continue to occur in hospitalised and recently discharged children; therefore, CDI testing is seldom appropriate in children with community-acquired disease.

When in doubt as to whether CDI testing is appropriate for a particular patient, consult colleagues with experience of CDI, such as microbiologists, gastroenterologists or infectious disease specialists.

3. **What is the appropriate testing strategy for patients fitting the case definition of suspected *C. difficile* infection?**

Appropriate sample types and pretesting sample storage conditions:

Formed stools submitted for CDI testing must be rejected by the laboratory. Transportation of stool samples to the laboratory and storage of samples prior to processing must be optimised to minimise delay and toxin degradation. Ideally, toxin testing must be performed within 2 h of sample collection. If there is a delay, samples can be stored at 4 °C for 3 days.^26^

There are various laboratory methods for CDI diagnosis (Table 2)^6^.

**TABLE 2:** Summary of available tests for *Clostridioides difficile* infection diagnosis.

Test	Sensitivity	Specificity	Target	Comments
Toxigenic culture (TC)	High	Low	*Clostridioides difficile* vegetative cells or spores followed by toxin gene	Reference standard
Nucleic acid amplification tests (NAAT)	High	Low/moderate	Toxin-encoding genes	High sensitivity is associated with a good NPV. Low specificity coupled with endemic CDI prevalence rates results in suboptimal PPV.
Glutamate dehydrogenase (GDH)	High	Low†	*Clostridioides difficile* common antigen	Screening test. For use as part of a testing algorithm.
Cell culture cytotoxicity neutralisation assay (CCCNA)	High	High	Toxins A and B	Reference standard
Toxins A and B enzyme immunoassays	Low/moderate	High	Toxins A and B	Not appropriate as stand-alone test for CDI diagnosis.

*Source:* Adapted from McDonald LC, Gerding DN, Johnson S, et al. Clinical practice guidelines for Clostridium difficile infection in adults and children: 2017 update by the Infectious Diseases Society of America (IDSA) and Society for Healthcare Epidemiology of America (SHEA). Clin Infect Dis. 2018 Mar 19;66(7):e1–e48.

CDI, *Clostridioides difficile* infection; NPV, negative predictive value; PPV, positive predictive value.

† , Lateral flow assays that combine detection of GDH and toxins A and B are available.

There are two reference standards for the laboratory diagnosis of CDI: cell culture cytotoxicity neutralisation assay (CCCNA) and toxigenic culture (TC).^27,28,29,30,31,32,33^ Cell culture cytotoxicity neutralisation assay detects toxin from stool filtrates. The reported sensitivity of CCCNA varies from 65% to 90% and depends on laboratory methodology and the comparator method. For TC, *C. difficile* is cultured from the stool sample and this is followed by detection of toxin-encoding genes. A positive CCCNA result is considered to represent a true CDI case (CCCNA is associated with a high PPV). Toxigenic culture has a superior NPV compared to CCCNA. However, TC has a lower PPV for true CDI, as positive TC results can reflect carriage of toxin-gene encoding *C. difficile* that is not currently producing toxin. Neither of these methods is suitable for CDI diagnosis in routine clinical laboratories as these methods are time-consuming, labour-intensive and require technical expertise.

The available NAAT assays provide a rapid and sensitive detection method for toxin-gene encoding *C. difficile*.^27^ The specificity of NAAT can be suboptimal. Glutamate dehydrogenase test is not as sensitive as NAAT but it is cheaper and simpler to perform. Sensitivities of > 90% for GDH have been reported in most evaluations.^34,35^ Currently available toxin IAs provide the high specificity required for the laboratory diagnosis of CDI but have suboptimal sensitivities.^27,36^

There are a number of studies evaluating at real time PCR cycle threshold (*C*_t_) correlation with toxin detection (toxin IA and/or cytotoxicity assay) for ‘preliminary diagnosis’ of CDI.^37,38,39^ The *C*_t_ may predict toxin IA positivity. However, further studies are needed to validate the analytical accuracy of *C*_t_ for toxin detection prediction. The best cut-offs can be determined based on the prevalence of CDI and the preferred sensitivity versus specificity. Individual laboratories need to determine the *C*_t_ for the local NAAT assay used and sample processing factors. Toxin testing is still warranted because of suboptimal correlation and correlation with clinical disease is also required.

The published evidence to guide selection of optimal laboratory testing strategy is generally of low quality. This is largely because of the lack of a standardised reference testing method and the absence of correlation of laboratory results with clinical disease.^40^ Samples must be collected prior to initiation of CDI therapy to avoid false-negative results.^41^

An algorithmic approach is recommended (Figure 1):

1. Glutamate dehydrogenase plus toxin A/B IA followed by NAAT for GDH-positive toxin-negative samples OR
2. Nucleic acid amplification test followed by toxin A/B IA for NAAT-positive samples.

Screening of samples commences with assays that are highly sensitive to the detection of *C. difficile* (NAAT or GDH). The high NPV of these tests allows for ruling out of CDI in samples testing negative. The detection of toxin A/B has high specificity for diagnosis of CDI in most settings. The detection of toxin is important for multiple reasons:

- Inappropriate selection of patients investigated – frequently clinically significant diarrhoea is not present or there is recent laxative use.^42^
- Shared risk factors for colonisation and infection with *C. difficile.*
- High rates of asymptomatic colonisation with *C. difficile.* Hospitalisation is associated with high rates of asymptomatic colonisation, ranging from 3% to 21%. This can increase to > 50% with prolonged hospitalisation.^43^
- Lack of clinical features that can reliably distinguish CDI from other infectious and non-infectious causes of diarrhoea. Nosocomial diarrhoea is frequently non-infectious in aetiology.^44^ *Clostridioides difficile* infection comprises 20% or less of infectious causes.
- The lower PPV of NAAT as a stand-alone test because of the prevalence of CDI in a non-outbreak setting.^34,45^ Stand-alone tests are unlikely to have an acceptable performance unless the prevalence of CDI is very high (> 40%).

Samples testing NAAT-positive toxin-negative may reflect false-negative toxin A/B results because of suboptimal sensitivity of toxin IAs (lower sensitivity than CCNA/cytotoxicity assays) or toxin degradation. However, this appears to more frequently represent asymptomatic colonisation.^42,45^ Further research is required to better define the optimal management of patients with NAAT-positive toxin-negative results.

The pretest probability of CDI must be considered when deciding whether treatment is indicated in these patients.^46^ Some studies have shown that NAAT-positive (or TC-positive) toxin-negative patients are clinically diverse from toxin-positive patients.^45,47,48,49^ The former group have been reported to have fewer cases of prolonged diarrhoea and a lower number of CDI-related complications and deaths. When compared to NAAT-negative patients, NAAT-positive toxin-negative patients have been shown to have similar inflammatory markers and 30-day mortality rates.^50^

Other studies, although with some methodological limitations, have shown that stand-alone NAAT-based testing is more sensitive than toxin IA and/or cytotoxicity assays and can also have a reasonable PPV when compared to the clinical diagnosis of CDI.^51,52^ The absence of toxin detection in TC/NAAT-positive samples may not be predictive of CDI severity.^53^

Stool multiplex NAAT assays for diarrhoeal pathogens often include *C. difficile*. If results from such testings detect *C. difficile*, the pretest probability of CDI must be considered and clinical consultation to determine the need for toxin testing is indicated.

If the results of stand-alone NAAT testing for *C. difficile* are positive, it is imperative that the report clearly states that only toxigenic *C. difficile* has been detected and that actual testing for free toxin has not been performed. Similarly, with algorithmic testing, for samples that test GDH positive, toxin negative and PCR positive, the report should clearly indicate that toxigenic *C. difficile* has been detected in the absence of toxin. When the testing platform used specifically detects the presence or absence of a hypervirulent strain (NAP 1/ribotype 027 or other), this must be reported.

4. **How should disease severity be graded?**

The use of severity grading scores assists in identifying patients who may benefit from aggressive treatment selection and early patient management decisions. Severity scores may also predict outcomes.

The severity of CDI ranges from asymptomatic carriage to mild diarrhoea to fulminant colitis and death (see Table 3)^6^.

**TABLE 3:** *Clostridioides difficile* infection severity grading.

Disease severity	Parameters
Mild to moderate	WBC ≤ 15 × 10^9^/L and
	Serum creatinine <133 *µ*mol/L
Severe	WBC > 15 × 10^9^/L and/or
	Serum creatinine ≥ 133 *µ*mol/L without criteria of fulminant disease
Fulminant	Hypotension or Ileus or Megacolon

*Source:* McDonald LC, Gerding DN, Johnson S, et al. Clinical practice guidelines for Clostridium difficile infection in adults and children: 2017 update by the Infectious Diseases Society of America (IDSA) and Society for Healthcare Epidemiology of America (SHEA). Clin Infect Dis. 2018 Mar 19;66(7):e1–e48.

WBC, White blood cells.

Several grading systems are described in the literature, but many are not validated and are based on expert opinion. The IDSA/SHEA guidelines severity scoring criteria (Table 3), which are based on expert opinion, are recommended for use in South Africa.^6^ These criteria, although not validated, have shown to have good NPV. A retrospective study compared the IDSA criteria with other criteria and found them to be reliable in predicting patient outcomes.^54^

Other studies have also shown white blood cell count and creatinine to be good predictors of mortality or complications in patients with CDI.^55,56^ However, in patients with concomitant haematological malignancies or renal dysfunction, these parameters are less useful.^57,58^

Other frequently described parameters for predicting the severity of disease are advanced age and hypoalbuminaemia.^54,58^

5. **What is the definition of recurrence?**

Individuals who meet the CDI case definition (including return of diarrhoeal stools with a positive laboratory test) after completion of CDI treatment, and who have new onset of symptoms between 2 and 8 weeks after the onset of symptoms from a previous episode of CDI.^59^

Most cases of rCDI occur in the first 2 weeks following completion of treatment of the initial episode. However, recurrences may occur up to 3 months following treatment. Following an initial episode of CDI, the risk of a recurrence ranges between 10% and 30%.^5,60^ Once a patient has one recurrence, the risk for further recurrences is between 40% and 60%.

Recurrent *C. difficile* infection is frequently because of relapse of previous infection rather than acquisition of a new strain.^61,62^

Risk factors for rCDI include:

- age > 65 years
- use of additional antibiotics after discontinuation of therapy for the initial CDI episode
- chronic renal failure
- severe or fulminant underlying illness
- previous fluoroquinolone use
- use of proton pump inhibitors after initial CDI episode
- inadequate immune response (evidence for an impaired immune response comes from small studies).^5,63^

Diagnosis of rCDI in patients with IBD is challenging (distinguishing flare from rCDI). Possible risk factors of recurrent disease in the IBD patient population (in addition to antibiotic exposure) include the use of 5-aminosalicylic acid, steroids and certain biologicals, but there are conflicting reports.^64^

6. **What is the role of repeat testing, following an initial negative test result, for suspected recurrence and for test of cure?**

Testing within 7 days of an initial negative result is not recommended unless there is a change in the clinical picture increasing the suspicion for CDI. The role of repeat testing is partly dependent on the sensitivity of the initial test performed; thus, the higher the sensitivity, the less the value of repeating. The high sensitivity of GDH and NAAT-based assays is associated with a good NPV.^35^ There are several studies demonstrating that where testing was repeated between 7 and 14 days following an initial negative test, the additional yield of positive results is only 1% – 3%.^65,66,67^

Testing is indicated for suspected rCDI and should include the detection of free toxin (using EIA or CCCNA). Empiric therapy for suspected recurrence is not recommended. Unwarranted treatment may further harm or disrupt gut microbiome.^68^ Post-infectious functional abdominal symptoms are common following an episode of successfully treated CDI.^69^ In addition, *C. difficile* colonisation commonly persists after an initial infection.^70^ Hence, the detection of free toxin is important for the diagnosis of recurrence.

Repeat testing for test of cure is not indicated. Many patients remain colonised with *C. difficile* and alterations of the gut microbiota persist following a successful treatment of CDI.^70,71^

### B. Treatment of initial episode of *Clostridioides difficile* infection in adults

#### Summary of recommendations

7. **What are the important supportive treatment strategies for *Clostridioides difficile* infection?**

**Recommendation:** Precipitating/implicated antibiotics must be stopped as soon as possible (*strong recommendation, moderate quality of evidence*).

8. **What are the recommended treatment options for an initial non-severe episode of *Clostridioides difficile* infection?**

**Recommendation:**

- Vancomycin 125 milligrams (mg) orally 6 hourly for 10 days

or

- Fidaxomicin 200 mg orally 12 hourly for 10 days (*strong recommendation, high quality of evidence*) (Table 4).
- Alternatively, if the above are not available, for example, in resource-limited settings: Metronidazole 500 mg orally 8 hourly for 10 days (*weak recommendation, high quality of evidence*).

**TABLE 4:** Recommendations for the treatment of *Clostridioides difficile* infection in adults.

*Clostridiodes difficile* infection episode	Recommended treatment
Initial non-severe CDI	Vancomycin 125 mg orally 6 hourly for 10 days
	Fidaxomicin 200 mg orally 12 hourly for 10 days
	If the above options are not available, then use metronidazole 500 mg orally 8 hourly for 10 days
Initial severe CDI	Vancomycin 125 mg orally 6 hourly for 10 days
	Fidaxomicin 200 mg orally 12 hourly for 10 days
Initial fulminant CDI	Vancomycin 500 mg orally 6 hourly or via nasogastric tube and intravenous metronidazole 500 mg every 8 h. If ileus present, consider adding rectal vancomycin 500 mg in 100 mL normal saline 6 hourly as retention enema.
Recurrent CDI: first and second recurrence	Fidaxomicin 200 mg orally 12 hourly for 10 days (if vancomycin was used for the initial episode)
	Vancomycin 125 mg orally 6 hourly for 10 days (if metronidazole was used for the initial episode)
	Vancomycin prolonged tapered and pulsed regimen (if a standard regimen of vancomycin was used for the initial episode)
Recurrent CDI: third and subsequent recurrence	Faecal microbiota transplant
	Fidaxomicin 200 mg orally 12 hourly for 10 days
	Vancomycin prolonged tapered and pulsed regimen

CDI, *Clostridioides difficile* infection.

9. **What are the recommended treatment options for an initial severe episode of *Clostridioides difficile* infection?**

**Recommendation:**

- Vancomycin 125 mg orally 6 hourly for 10 days

or

- Fidaxomicin 200 mg orally 12 hourly for 10 days (*strong recommendation, high quality of evidence*) (Table 4).

10. **What are the recommended treatment options for an initial fulminant episode of *Clostridioides difficile* infection?**

**Recommendation:**

- Vancomycin 500 mg orally every 6 hourly or via nasogastric tube (*strong recommendation, moderate quality of evidence*).
- If ileus present, then consider adding rectal vancomycin 500 mg in 100 millilitre (mL) normal saline 6 hourly as retention enema (*weak recommendation, low quality of evidence*) and intravenous metronidazole 500 mg every 8 hourly (*strong recommendation, moderate quality of evidence*) (Table 4).

11. **Should empiric antibiotic therapy be considered in all patients if there is an anticipated delay in diagnosis?**

**Recommendation:** Empiric therapy for CDI should be considered in patients with mild-to-moderate disease severity only if there is an anticipated delay in diagnosis of > 48 h. In severe and fulminant CDI, initiation of empiric therapy is urgent and is not dependent on the results of laboratory tests (*weak recommendation, low quality of evidence*).

**Rationale for recommendations**

7. **What are the important supportive treatment strategies for *Clostridioides difficile* infection?**

When possible stopping of the antibiotic(s) associated with the CDI episode is recommended. Continuation of antibiotics is associated with poorer clinical responses and increased risk of rCDI.^72^

8. **What are the recommended treatment options for an initial non-severe episode of *Clostridioides difficile* infection?**

Oral metronidazole or vancomycin has traditionally been the treatment options for CDI.

Oral metronidazole is cheaper and is assumed to be associated with lower vancomycin-resistant enterococci (VRE) selection risk. In previous guidelines, metronidazole was recommended as a first-line treatment for non-severe CDI and vancomycin as the first choice for severe CDI.^73,74^ Since these publications, results from large, multicentre RCTs have demonstrated that metronidazole is inferior to vancomycin in the treatment of CDI.^6,75,76^

Data from three randomised controlled trials showed inferior response rates (RRs) at the end of treatment with metronidazole compared to vancomycin, with a response rate of 0.89 (95% confidence interval [CI] 0.82–0.96; *p* = 0.002). Metronidazole was also found to be inferior to vancomycin for sustained response at 21–30 days after treatment (RR 0.84; 95% CI 0.74–0.94; *p* = 0.002). Combined, RCTs published since 2000 demonstrated that metronidazole is inferior to oral vancomycin for clinical cure in patients with CDI (*p* < 0.006).^6,75,76^ A recent retrospective study of hospitalised patients with mild-to-moderate CDI found that metronidazole was inferior to vancomycin for treatment response in this group of patients as well.^77^

Data from two RCTs also showed similar response rates at the end of treatment with fidaxomicin and vancomycin, (RR 1.09; 5% CI 0.98–1.1; *p* = 0.36).

Fidaxomicin was superior to vancomycin for sustained response at 25 days (RR 1.2; 95% CI 1.1–1.4; *p* < 0.001).^78^

A recent retrospective review and meta-analysis also found that compared with vancomycin, fidaxomicin is associated with superior sustained clinical response rates (odds ratio [OR] 0.67; 95% CI 0.55–0.82) for recurrence.^79^

The use of oral metronidazole should be restricted to an initial episode of non-severe CDI or in cases where other therapies are not available.

##### Duration of therapy

Most RCTs have compared 10-day regimens of CDI treatment agents, and it appears that a 10–day period is sufficient to resolve symptoms in most patients. If patients have improved, but do not have symptom resolution by 10 days, an extension of treatment duration to 14 days should be considered.^74^

There is currently no evidence to suggest that a shorter duration of therapy could lead to higher recurrence rates and thus further research is required.^80^ It is highly recommended to adhere to generally accepted dosage regimens of currently used agents.

9. **What are the recommended treatment options for an initial severe episode of *Clostridioides difficile* infection?**

The use of high doses of vancomycin (500 mg orally four times daily) was previously recommended by some guidelines for the management of severe complicated CDI.^74^ However, there is insufficient evidence to support the use of doses > 125 mg four times daily in the absence of ileus.^81^

10. **What are the recommended treatment options for an initial episode of fulminant *Clostridioides difficile* infection?**

Vancomycin at high doses has previously been recommended for fulminant CDI, despite the lack of high-quality evidence.

In the presence of ileus, vancomycin can also be given rectally although it remains unclear whether adequate concentrations of the drug are reached beyond the left colon.^6^

Despite the lack of data, it seems reasonable to administer oral and/or rectal vancomycin at higher doses for patients with fulminant CDI (500 mg 6 hourly orally and 500 mg in 100 mL of normal saline by retention enema). The use of high-dose vancomycin is safe, but it is appropriate to monitor serum trough concentrations to rule out drug accumulation.^82^

Intravenous metronidazole (500 mg 8 hourly) should be used in addition to vancomycin.^83^ This is particularly important in the presence of ileus as intravenous metronidazole may achieve therapeutic concentrations in an inflamed colon.

In patients not responding to vancomycin and metronidazole, intravenous tigecycline (loading dose of 100 mg followed by 50 mg twice daily) has been used as adjunctive or alternative therapy, but no RCTs have been performed to date.^84^ Recently published retrospective studies have shown conflicting results regarding the efficacy of tigecycline for fulminant CDI.^85,86,87,88^ Because of the poor outcomes associated with fulminant CDI, there may be a role for adjunctive tigecycline.^84^ Consideration of surgical intervention and faecal microbiota transplantation (FMT) in such settings is also indicated.

Surgical intervention can be life-saving in patients with fulminant disease; timely consultation with a gastroenterologist and surgeon is critical in such clinical circumstances.

11. **Should empiric antibiotic therapy be considered in all patients if there is an anticipated delay in diagnosis?**

A guiding principle of infectious disease management is that specimens for diagnosis should be collected before initiation of therapy whenever possible.^41^ Empiric therapy for CDI may result in false-negative diagnostic test results.

Empiric therapy for CDI should be considered in all patients with mild-to-moderate disease severity if there is an anticipated delay of > 48 h or if a patient presents with severe or fulminant CDI.^6^ For all other patients, antibiotic therapy should be started after diagnosis to limit the overuse of antibiotics and the associated toxicities, which include overgrowth of multidrug-resistant pathogens.^41^

### C. Treatment of recurrent *Clostridioides difficile* infection in adults

#### Summary of recommendations

12. **What are the optimal treatment regimens for recurrent *Clostridioides difficile* infection? (First and second recurrence)**

**Recommendation:**

- Fidaxomicin 200 mg orally 12 hourly for 10 days (if vancomycin was used for the initial episode) (*weak recommendation, moderate quality of evidence*)

or

- Vancomycin 125 mg orally 6 hourly for 10 days (if metronidazole was used for the initial episode) (*weak recommendation, moderate quality of evidence*)

or

- Vancomycin prolonged tapered and pulsed regimen (if a standard regimen of vancomycin was used for the initial episode) (*weak recommendation, low quality of evidence*) (Table 4).

13. **What are the optimal treatment regimens for recurrent *Clostridioides difficile* infection? (Third and subsequent recurrences)**

**Recommendation:**

- Faecal microbiota transplant (*strong recommendation, high quality of evidence*)

or

- Fidaxomicin 200 mg orally 12 hourly for 10 days (*weak recommendation, low quality of evidence*)

or

- Vancomycin prolonged tapered and pulsed regimen (*weak recommendation, low quality of evidence*).

14. **What is the role of bezlotoxumab in the management of recurrent *Clostridioides difficile* infection?**

Bezlotoxumab is not yet licensed for use in South Africa.

##### Rationale for recommendations

**12 and 13. What are the optimal treatment regimens for recurrent *Clostridioides difficile* infection?**

The management of rCDI is the same for relapse (with same strain from prior CDI episode) and re-infection (with different strain).

Recurrence rates are significantly lower following initial treatment with fidaxomicin compared to vancomycin.^78,89^

A first or second recurrence may be treated with fidaxomicin or a tapered and pulsed regimen of vancomycin. In a randomised, stratified sub-study of patients with a first recurrence, a subsequent second recurrence at 28 days was less common following therapy with fidaxomicin compared to a standard 10-day course of vancomycin (19.7% vs. 35.5%; *p* = 0.045).^90^ There is limited published evidence on the efficacy of fidaxomicin for multiply rCDI. A small multicentre retrospective review showed that the efficacy of fidaxomicin for the prevention of rCDI is reduced in multiply rCDI compared to that in initial and first recurrence episodes.^91^ This is expected as patients with multiply rCDI are likely to have severe gut dysbiosis.

Various vancomycin tapered and pulsed regimens have been described in the literature. One such regimen recommended by IDSA advises the following: after a 10–14-day course of vancomycin 125 mg four times a day, give vancomycin 125 mg twice daily for a week, 125 mg once daily for a week and then 125 mg every 2–3 days for 2–8 weeks.^6^ There are only a few published studies evaluating the efficacy of tapered and pulsed vancomycin treatment regimens for rCDI.^70,92,93,94^ Tan and Johnson suggested that close patient follow-up and individualisation of tapered and pulsed vancomycin regimens can be associated with favourable outcomes.^94^ Optimal treatment for rCDI (OpTION trial) (NCT02667418) is a randomised clinical trial currently underway. The study has 3 arms, comparing: (1) standard fidaxomicin therapy (10-day course) to (2) standard vancomycin therapy (10-day course) that is followed by tapered and pulsed vancomycin (31 days of vancomycin overall) and (3) standard vancomycin therapy (10-day course) alone, for sustained response at 59 days.^95^

The rationale for the FMT recommendation is discussed in section D (The role of FMT in the treatment of CDI in adults).

14. **What is the role of bezlotoxumab in the management of recurrent *Clostridioides difficile* infection?**

Bezlotoxumab is a fully human monoclonal antibody that binds to and neutralises *C. difficile* toxin B.^96^ This product is not yet licensed in South Africa. Two double-blind, randomised, placebo-controlled, phase-3 studies (A study of MK-3145, MK-6072 and MK-3415A in participants receiving antibiotic therapy for *C. difficle* infection [MODIFY I] and A study of MK-6702 and MK-3415A in participants receiving antibiotic therapy for *C. difficile* infection [MODIFY II]) investigated the efficacy of bezlotoxumab in the prevention of rCDI in adults with primary or rCDI who were receiving standard of care antibiotics. The difference in the rate of sustained cure (initial clinical cure of baseline CDI episode and the absence of recurrent infection for 12 weeks) between bezlotoxumab and placebo was statistically significant in MODIFY II and pooled MODIFY I and II results but not in MODIFY I.^97^

The rate of rCDI was lower with bezlotoxumab compared to placebo in patients who had at least one of five predefined risk factors (age ≥ 65 years, previous CDI in last 6 months, immunocompromised, severe CDI and infection with a hypervirulent strain) for rCDI or for adverse outcomes related to CDI. Patients with three or more of these risk factors derived the optimum benefit.^98^ For patients infected with a hypervirulent strain, the reduction in rCDI was not statistically significant. There was no benefit in patients who did not have any of the mentioned risk factors. Fidaxomicin was used in only 4% of patients (the majority were treated with metronidazole or vancomycin) and thus the effects of bezlotoxumab use with fidaxomicin warrant further investigation. Serious adverse events related to congestive cardiac failure (CCF) were reported more frequently in the bezlotoxumab group.^98^ Whilst deemed to be an artefact by the European Medicines Agency, the reasons for the difference in CCF numbers between the bezlotoxumab and placebo groups are under investigation.

The efficacy of bezlotoxumab in special patient populations regarded as being at increased risk of rCDI requires further investigation. Some of the currently available data of post hoc analyses, with the studies not intended to show statistical significance between the groups, are as follows:

Inflammatory bowel disease: small number of IBD patients included in the study. The trend is towards reduced rCDI in the bezlotoxumab group (an absolute reduction of 27.2%; 95% CI −57.9 to 9.6).^99^

Concomitant antibiotics: bezlotoxumab may be associated with a reduction in rCDI in patients receiving concomitant antibiotics for other infections during initial CDI treatment or in the 90 days following treatment for initial CDI episode.^100^

Haematologic malignancies and solid organ tumours: lower rate of rCDI in haematologic malignancies and solid organ transplant but small patient numbers. It requires further investigation.^101^

Renal dysfunction: reduced rCDI rate in bezlotoxumab group (absolute reduction −17.1%; 95% CI −23.4 to −10.6).^102^

### D. The role of faecal microbiota transplant in the treatment of *Clostridioides difficile* infection in adults

#### Summary of recommendations

15. **Should faecal microbiota transplant be used as a first-line therapy for an initial episode of *Clostridioides difficile* infection?**

**Recommendation:** At present, the routine use of FMT cannot be recommended as a first-line therapy for an initial episode of CDI (*strong recommendation, moderate quality of evidence*).

16. **Should faecal microbiota transplant be used as a treatment strategy in recurrent *Clostridioides difficile* infection?**

**Recommendation:** Faecal microbiota transplant is recommended as the first-line therapy for third and subsequent recurrences (*strong recommendation, high quality of evidence*) (Table 4).

17. **Is faecal microbiota transplantation a safe and effective therapy in patients who are immunocompromised or on immunosuppressive medications (special groups)?**

**Recommendation:** From the limited data available, it appears that FMT is safe and effective for the treatment of rCDI in patients with HIV infection (regardless of CD4 count), malignancy and chemotherapy, solid organ transplant, chronic kidney disease and patients receiving haemodialysis (*weak recommendation, low quality of evidence*).

**Rationale for recommendations**

15. **Should faecal microbiota transplant be used as a first-line therapy in *Clostridioides difficile* infection?**

Disruption of the host gut microbiota profile, also called dysbiosis, leads to a decrease in gut diversity, resulting in *C. difficile* overgrowth. Transplantation of donor stool in the host, called FMT, aims to restore the host gut microbiota profile to normal, with resolution of the infection. The first RCT on FMT was published in 2013: FMT following antibiotic treatment with an oral glycopeptide was reported to be highly effective in treating multiply rCDI.^103^

Only a handful of reports have assessed FMT as a first-line treatment strategy, and although a few have shown positive results, the total number of patients treated was small. The overall evidence supports medical therapy as the first-line treatment.^104^ The only randomised trial evaluating FMT versus vancomycin for initial CDI found vancomycin superior, with fewer failures and recurrences.^105^ Currently, there is insufficient data regarding the long-term sequelae (including the possibility of malignant, autoimmune, metabolic or neuropsychiatric disorders) of FMT.

16. **Should faecal microbiota transplant be employed as a treatment strategy in recurrent *Clostridioides difficile* infection?**

Faecal microbiota transplant is recommended as a first-line therapy in multiply rCDI, regardless of severity.

Many randomised controlled studies, cohort studies and case reports have confirmed the benefits of FMT in patients with rCDI. In an RCT, Cammarota et al. showed that FMT is superior in rCDI compared to vancomycin alone for the resolution of CDI (90% vs. 26%).^106^ A systematic review by Drekonja et al. showed that FMT resulted in the resolution of symptoms in > 85% of patients with rCDI compared to 31% in the vancomycin group.^107^ A systematic review and meta-analysis by Kassam et al. showed similar resolution rates of 89% for FMT as treatment for rCDI.^108^ A recent, larger, double-blind RCT study confirmed resolution rates above 80% for FMT in rCDI.^109^

In conclusion, evidence for FMT as a treatment modality in rCDI is well established, with high (> 85%) success rates in several RCTs. The role of preinfusion antibiotics (vancomycin or fidaxomicin) in the setting of rCDI is not clear. This needs prospective randomised investigation. The European consensus conference on FMT in clinical practice guideline recommends treatment with vancomycin or fidaxomicin for a minimum of 3 days before FMT and the stopping of antibiotics 12–48 h before FMT.^110^

There is some evidence that the route of FMT delivery has an impact on the efficacy of the treatment. A systematic review and meta-analysis of observational and RCTs published in 2016 found a difference in cure rates between lower gastrointestinal and upper gastrointestinal delivery – 95% and 88%, respectively (*p* = 0.02).^111^ Analysis limited to cure rates with a single FMT infusion did not show a significant difference related to delivery route. Findings from two other meta-analyses suggested that CDI resolution rates are lower with FMT delivered by enema compared to colonoscopy.^112,113^

Repeated FMT infusions following failure of response to an initial FMT are associated with an incremental success rate.^111^

17. **Is faecal microbiota transplantation a safe and effective therapy in patients who are immunocompromised or on immunosuppressive medications (special groups)?**

There are no RCTs in special patient groups. Recommendations in patients with HIV, malignancies, solid organ transplants and chronic kidney disease are based on case reports and case series with small patient numbers.

The available evidence suggests that FMT is safe and effective in the patient populations described here.^111,114,115,116^

Recommendations for FMT in the IBD population are discussed in section E (Treatment of CDI in special risk populations, including IBD). Recommendations for FMT in the paediatric population are discussed in section F (Treatment of CDI in the paediatric population).

For FMT donor screening and procedure, refer to the European consensus conference on faecal microbiota transplantation in clinical practice.^110^

### E. Treatment of *Clostr idioides difficile* infection in special risk populations, including inflammatory bowel disease

#### Summary of recommendations

18. **What are the optimal treatment regimens for *Clostridioides difficile* infection in special risk populations (excluding inflammatory bowel disease)?**

**Recommendation:** Treatment of CDI in special risk populations is similar to general treatment guidelines and is guided by disease severity.

19. **What is the recommended treatment for *Clostridioides difficile* infection in inflammatory bowel disease?**

**Recommendation:** In general, the treatment of CDI in IBD is similar to that of non-IBD patients. The treatment of an initial episode of CDI in adults is discussed in section B. The treatment of rCDI in adults is discussed in section C. For fulminant CDI in IBD, early surgical consultation is recommended in addition to medical management.

**Rationale for recommendations**

18. **What are the optimal treatment regimens for *Clostridioides difficile* infection in special risk populations?**

Small case numbers and the lack of high-quality data preclude this guideline from making specific recommendations in populations at risk.

***Clostridioides difficile* infection and human immunodeficiency virus**

*Clostridioides difficile* is amongst the most commonly isolated pathogens in HIV-infected patients with diarrhoeal illness and is greater than or equal to twofold more common in HIV-infected individuals.^117^ This association is stronger in those with low CD4 T-cell counts or those meeting clinical criteria for an acquired immunodeficiency syndrome (AIDS) diagnosis. The increased risk can be attributed in part to frequent hospitalisation and antimicrobial use, but HIV-related alterations in faecal microbiota, gut mucosal integrity and humoral and cell-mediated immunity likely also play a role.^117^

19. **What is the recommended treatment for *Clostridioides difficile* infection in inflammatory bowel disease?**

Patients with IBD, in particular, those with ulcerative colitis, are at increased risk of developing CDI.^20,118^ Higher colectomy rates, more extended hospital stays, increased mortality and hospital costs have been associated with CDI and concomitant IBD.^20,119,120,121^

Evidence is scarce to direct a specific antibiotic choice in CDI-IBD patients; however, treatment failure rates of up to 50% have been reported in IBD patients receiving metronidazole only.^122^ In addition, a single centre study managed to cut their colectomy rates from 45.5% to 25.0% 1 year after changing the first-line therapy from metronidazole to vancomycin.^123^ In CDI-IBD patients, oral vancomycin is the treatment of choice. Although Cornely and colleagues excluded IBD patients from their study, they showed that fidaxomicin was superior in preventing recurrence.^124^ Fidaxomicin is a reasonable alternative to oral vancomycin in CDI-IBD patients who are already at high risk of recurrence.

Treatment recommendations for severe and fulminant CDI-IBD are no different from CDI in non-IBD patients. It may, however, be necessary to withhold immunomodulators (IMM) in CDI-IBD patients who have been on maintenance IMM therapy prior to developing CDI. On the other hand, in CDI-IBD patients with severe colitis not responding to CDI treatment, one may need to consider adding steroids or a biological agent as colon salvage therapy. Individualised therapy is indicated for this patient subset. Surgical management is generally reserved for CDI-IBD refractory to medical therapy.

Immunosuppression in CDI-IBD is a challenging area that still causes significant disagreement amongst practising gastroenterologists.^125^ There is limited data to guide the use of immunomodulatory agents in IBD patients with CDI.^126^ The available data are of low quality and contradictory. Randomised controlled trials are therefore needed to investigate the optimal management approach to this clinical dilemma.

**Recurrent *Clostridioides difficile* infection in inflammatory bowel disease**

Inflammatory bowel disease patients are at higher risk of developing rCDI. A retrospective cohort of 503 patients showed that rCDI occurs in one-third of IBD patients.^64^ Although rCDI is relatively common in IBD, repeat CDI testing should be avoided in the absence of clear changes in the clinical presentation of suspected disease.^127^ Repeat testing to determine clinical cure is also not recommended as asymptomatic carriage can occur in up to 8.2% of patients with IBD, in particular, in patients with ulcerative colitis.^127,128^

As there is limited evidence to guide the management of rCDI in IBD, the treatment of rCDI in IBD should not differ from the general population.

**Faecal microbiota transplant in *Clostridioides difficile* infection and inflammatory bowel disease**

Faecal microbiota transplant appears to be safe and effective in the management of CDI in IBD and should be offered in rCDI. Faecal microbiota transplant is reported to be 70% – 90% effective in achieving cure after initial treatment and 89% – 98% effective for overall cure (following > 1 FMT).^129,130,131^ Adverse events noted in CDI-IBD patients who received FMT include worsening of IBD and hospitalisation, need for colectomy and superadded infection. Further well-designed studies are needed to investigate the pathogenesis of the IBD flare-ups reported in many retrospective reports.

### F. Treatment of *Clostridioides difficile* infection in the paediatric population

#### Summary of recommendations

20. **What are the recommended treatment options for an initial non-severe episode of *Clostridioides difficile* infection treatment in the paediatric population?**

**Recommendation:**

- Metronidazole 7.5 mg/kg/dose 8 hourly (maximum dose 500 mg) orally for 10 days

or

- Vancomycin 10 mg/kg/dose 6 hourly (maximum dose 125 mg) orally for 10 days (*weak recommendation, low quality of evidence*) (Table 5).

**TABLE 5:** Recommended treatment options for *Clostridioides difficile* infection in the paediatric population.

Clostridiodes difficile infection episode	Recommended treatment
Initial non-severe CDI	Metronidazole 7.5 mg/kg/dose 8 hourly (maximum dose 500 mg) orally for 10 days
	Vancomycin 10 mg/kg/dose 6 hourly (maximum dose 125 mg) orally for 10 days
Initial severe or fulminant CDI	Vancomycin 10 mg/kg/dose 6 hourly (maximum dose 500 mg) orally for 10 days. Consider the addition of metronidazole in fulminant cases. Vancomycin rectally for patients with ileus or those who are unable to tolerate oral therapy.
Recurrent non-severe CDI: first recurrence	Metronidazole (as for initial episode)
	Vancomycin (as for initial episode) if metronidazole used previously
Recurrent non-severe CDI: second and subsequent recurrence	Vancomycin, if metronidazole alone was used previously
	Vancomycin in a tapered and pulsed regimen. Vancomycin 10 mg/kg/dose 6 hourly (maximum dose 125 mg) orally for 10–14 days, then 12 hourly for 7 days, then daily for 7 days and then every 2–3 days for 2–8 weeks
Recurrent severe or fulminant CDI: first and subsequent recurrences	Treat as initial episode of severe/fulminant CDI

CDI, *Clostridioides difficile* infection.

21. **What are the recommended treatment options for an initial severe or fulminant episode of *Clostridioides difficile* infection in the paediatric population?**

**Recommendation:** Vancomycin 10 mg/kg/dose 6 hourly (maximum dose 500 mg) orally for 10 days. Consider the addition of metronidazole in fulminant cases. There may be a role for vancomycin rectally for patients with ileus or those who are unable to tolerate oral therapy (*weak recommendation, low quality of evidence*) (Table 5).

22. **What are the recommended treatment options for a first recurrence of non-severe *Clostridioides difficile* infection in the paediatric population?**

**Recommendation:**

- Metronidazole (as for initial episode)

or

- Vancomycin (as for initial episode) if metronidazole used previously (*weak recommendation, low quality of evidence*) (Table 5).

23. **What are the recommended treatment options for second and subsequent recurrences of non-severe *Clostridioides difficile* infection in the paediatric population?**

**Recommendation:**

- Vancomycin, if metronidazole alone was used previously

or

- Vancomycin in a tapered and pulsed regimen. Tapered and pulsed regimen: Vancomycin 10 mg/kg/dose 6 hourly (maximum dose 125 mg) orally for 10–14 days, then 12 hourly for 7 days, then daily for 7 days and then every 2–3 days for 2–8 weeks (*weak recommendation, low quality of evidence*) (Table 5).

24. **What are the recommended treatment options for a first and subsequent recurrence of severe or fulminant *Clostridioides difficile* infection in the paediatric population?**

**Recommendation:** Treat as for an initial episode of severe/fulminant CDI (*weak recommendation, low quality of evidence*) (Table 5).

25. **Is faecal microbiota transplant recommended for use in children and adolescents?**

**Recommendation:** Faecal microbiota transplant is not currently recommended for the treatment of CDI in the paediatric population in South Africa. In exceptional cases, seek subspecialist input before providing FMT (*weak recommendation, low quality of evidence*).

26. **What is the role of fidaxomicin for the treatment of *Clostridioides difficile* infection in the paediatric population?**

**Recommendation:** Fidaxomicin is not currently recommended – seek subspecialist input in exceptional cases (*weak recommendation, low quality of evidence*).

**Rationale for recommendations**

20. **What are the recommended treatment options for an initial episode of non-severe *Clostridioides difficile* infection in the paediatric population?**

Clinical studies on the treatment of CDI in the paediatric population are limited. The available evidence is derived from studies with weak study design and recommendations are made on generally low-quality evidence. Historically, both vancomycin and metronidazole have been recommended for the treatment of CDI in children and adolescents.^6,132^ Although paediatric CDI incidence is increasing, it remains very rare in infants and uncommon in young children. Colonisation with *C. difficile* is very common in infants and then declines to adult levels by approximately 3 years of age.^133^

The majority of CDI in children is mild to moderate in severity. Complications and mortality are uncommon even in children with severe disease regardless of the treatment provided.^6^

Metronidazole or vancomycin is recommended for the initial episode of non-severe CDI.

There are insufficient data to recommend vancomycin over metronidazole for initial mild disease. As there is limited high-quality evidence to guide the management of CDI in children, recommendations are extrapolated from adult data and expert opinion.^6^ There are no high-quality studies directly comparing these two agents in children.^6,133,134,135^ A small study found a non-significant higher rate of treatment failure with metronidazole.^132^

21. **What are the recommended treatment options for an initial episode of severe or fulminant *Clostridioides difficile* infection in the paediatric population?**

Oral vancomycin is recommended over metronidazole for the treatment of severe/fulminant paediatric CDI. The addition of intravenous metronidazole for fulminant disease must be considered. Rectal vancomycin with intravenous metronidazole may be considered in patients with severe/fulminant CDI if unable to tolerate oral therapy.

These recommendations are based on adult data and international guidelines where vancomycin is preferred to metronidazole as treatment failure is less common.^6,76,135^ The addition of intravenous metronidazole has been shown to improve clinical outcomes in critically ill adult patients.^74^ Although there are no data to support this practice in children, it is suggested because of the ability to achieve therapeutic metronidazole concentrations at the site of CDI where gut dysmotility may impair the delivery of oral medication in severe disease. For children with fulminant CDI, higher doses of vancomycin have been suggested (maximum 500 mg/dose) as theoretically severe colitis may result in systemic absorption and lower colonic luminal vancomycin levels.^136^ At present, sufficient data are not available to recommend this approach. The optimal dose and volume of rectal vancomycin are not well established.

22. **What are the recommended treatment options for a first recurrence of non-severe *Clostridioides difficile* infection in the paediatric population?**

Metronidazole or vancomycin is recommended for the first non-severe recurrence of paediatric CDI.

There are currently no robust clinical data to recommend one agent over the other for initial recurrence of CDI in the paediatric population. Thus, either metronidazole or vancomycin is recommended. Vancomycin is suggested if metronidazole was used initially.^6,132,134^

23. **What are the recommended treatment options for second and subsequent recurrences of non-severe *Clostridioides difficile* infection in the paediatric population?**

Vancomycin is recommended instead of metronidazole for the management of second and subsequent recurrences of paediatric CDI. Vancomycin may be used in a pulsed-tapered regimen if it was used previously or in a standard regimen if metronidazole was used previously.

The optimal regimen for the treatment of recurrent paediatric CDI is not known.

There are no convincing paediatric data that suggest either drug is more effective for multiple episodes of CDI.^132,134^ Pulsed-tapered vancomycin regimens have been used in children, extrapolating from experience in adults.^6,70,133^ Metronidazole has been linked to neurotoxicity when used in the treatment of rCDI and therefore is not recommended.^6^

24. **What are the recommended treatment options for a first and subsequent recurrence of severe or fulminant *Clostridioides difficile* infection in the paediatric population?**
  Treatment is as for an initial episode of severe or fulminant CDI. See question 21 for rationale.

25. **Is faecal microbiota transplant recommended for recurrent *Clostridioides difficile* infection in children and adolescents?**

There is a lack of controlled studies evaluating FMT for rCDI in children. However, published small case series and case reports show a high success rate.^137^ The North American and European Societies for Pediatric Gastroenterology, Hepatology and Nutrition have provided guidance on when to consider FMT for CDI in children.^138^

In the South African setting, it is recommended to refer children with a second CDI recurrence to a paediatric gastroenterologist or infectious disease specialist.

26. **What is the role of fidaxomicin in the treatment of paediatric *Clostridioides difficile* infection?**

Fidaxomicin has not been extensively studied in children; however, preliminary data are encouraging.^139^ A phase-3 multicentre study to investigate the safety and efficacy of fidaxomicin (oral suspension or tablets) and vancomycin (oral liquid or capsules) in pediatric subjects with Clostridium difficile-associated disease (SUNSHINE) evaluated the safety and efficacy of fidaxomicin and vancomycin for paediatric CDI and the results demonstrated a trend for improved efficacy outcomes with fidaxomicin compared with vancomycin.

Currently, fidaxomicin is not recommended for use in children; however, as more data emerge, this recommendation may change.

### G. Surveillance of *Clostridioides difficile* infection

#### Summary of recommendations

27. **How should surveillance of *Clostridioides difficile* infection be conducted and reported at a facility level?**

**Recommendation:** To enable tracking and comparison of CDI rates within the facility, currently recommended CDI surveillance definitions, including cases, infection origin and rates, should be used (*strong recommendation, high quality of evidence*).

28. **What is the minimal level of *Clostridioides difficile* infection surveillance required?**

**Recommendation:** At a minimum, facilities should conduct laboratory-based surveillance of healthcare facility-onset (HO) CDI and monitor CDI using incidence risk (new cases/10 000 admissions) or incidence density (new cases/10 000 inpatient days) (*strong recommendation, low quality of evidence*).

29. **What is the facility versus laboratory obligation/responsibility in terms of reporting (notifiable medical condition requirements)?**

**Recommendation:** Facilities are not required to directly report CDI to the notifiable medical condition (NMC) system. This is the responsibility of the laboratory identifying the pathogen.

##### Rationale for recommendations

27. **How should surveillance of *Clostridioides difficile* infection be conducted and reported at a facility level?**

Although CDIs have been considered nuisance infections amongst people exposed to healthcare, the epidemiology of this pathogen is changing globally. Over the last decade, the incidence of CDI has increased in European, Australian and North American countries.^14,124,140^ The severity of disease and CDI-associated mortality has also increased, presumably because of the emergence and dissemination of the hypervirulent *C. difficile* strain ribotype 027 or NAP1.^124,141,142^ Recently, *C. difficile* was shown to be an equally significant pathogen amongst hospitalised patients in Asian countries, although the prevalence of the hypervirulent strain was considerably lower compared to Western countries.^14^ In addition, community-acquired *C. difficile* infections amongst individuals with no healthcare exposure have now increased, accounting for almost half of all infections in some settings.^18^

Despite the emergence of *C. difficile* as a major public health threat, its burden in most developing countries remains unknown.^143,144^ In South Africa, rates and the burden of *C. difficile* are unknown, with limited single-centre studies showing a prevalence of 9.2% – 22.0% amongst patients with diarrhoeal illnesses, and an incidence of 8.7 cases per 10 000 admissions reported for one facility.^8,127,145^ Amongst these studies, two that reported CDI origin showed that healthcare-associated CDI occurred more frequently than community-associated infections, suggesting that CDI is more of a concern amongst hospitalised patients in South Africa.^8,127^ Because of the lack of reports, it is unclear whether South African facilities are monitoring CDIs and whether this is an important pathogen in this setting.

The use of standardised surveillance methods and definitions allows for effective monitoring of the occurrence and spread of health threats, detection of outbreaks and monitoring of interventions. Because of the emergence of *C. difficile* in the last decade, the National Health Safety Network (NHSN) of the CDC and the European Centre for Disease Control and Prevention (ECDC) have published CDI surveillance guidelines, which have been widely adopted to monitor this pathogen.^59,146,147^ A case is defined as a patient with diarrhoea or toxic megacolon and a positive laboratory test result for *C. difficile* toxin A and/or B, or detection of a toxin-producing *C. difficile* organism by culture or other means, or pseudomembranous colitis diagnosed by endoscopic examination or surgery, or pseudomembranous colitis diagnosed by histopathology. In settings where laboratory testing is only done on loose stools, the laboratory criterion can be used for routine surveillance. For defining the origin of CDI, time and location at symptom onset or specimen collection as well as previous history of healthcare exposure are used. Healthcare facility-onset (HO) cases are defined as symptom onset or specimen collection > 3 days after admission. Community-onset (CO) cases are defined as symptom onset or specimen collection ≤ 3 days after admission, and community-onset healthcare facility-associated (CO-HCFA) cases are defined as symptom onset or specimen collection ≤ 3 days from admission, and previous admission or discharge from a healthcare facility in the last 28 days. Rates of CDI can be expressed using different denominators. Typically, the number of cases (numerator) occurring over the surveillance period is divided by patient days (sum of the number of days each patient had an overnight stay), admissions (sum of all patients with an overnight stay), discharges (sum of all patients discharged) or bed-days (number of hospital beds multiplied by the surveillance period). For HO-CDI, incidence density (i.e. cases per 10 000 patient days) is the most beneficial as it provides risk information of *C. difficile* transmission per day whilst admitted and allows for comparisons between facilities or wards, which may admit patients requiring different treatments and thus length of stay. However, alternative denominators may be used depending on availability or feasibility. For CO-CDI, healthcare exposure is not a risk factor and alternative denominators may be used. As rates are determined using only incident cases, rCDI episodes must be excluded from the numerator. Recurrent cases are defined as patients with subsequent episodes > 14 days and ≤ 56 days following the initial symptoms onset or specimen collection.

28. **What is the minimal level of *Clostridioides difficile *infection** surveillance required?**

In resource-limited settings, such as low-income countries, surveillance is challenging because of problems with understaffing, lack of skilled and knowledgeable personnel, lack of laboratory diagnostic capacity and lack of national guidelines.^148,149^ It is therefore important that facilities in resource-limited settings individualise *C. difficile* surveillance, selecting components suitable to available resources. As exposure to healthcare is the most recognisable risk factor for CDI, surveillance in healthcare facilities should primarily monitor hospital-onset infections. This will allow tracking and detection of increases in the number of CDI cases.^150^ The European *Clostridium difficile* Infection Surveillance Network (ECDIS-Net) conducted a study to assess different methods of surveillance and showed that with ‘Minimal’ surveillance, which imposed the least amount of workload through collection of only aggregated numerator and denominator data, incidence rates could be determined and tracked.^146^ However, the ‘Light’ and ‘Enhanced’ surveillance methods where additional epidemiologic case information was collected, including CDI origin, microbiological and outcome data, offered more detailed information that would allow for targeted control measures, although with more workload. Facilities may also elect to perform only laboratory-based surveillance as this approach allows for diagnostic data to be used as a proxy for clinical infection surveillance and is less labour intensive than using clinical data to define cases.^147^

29. **What is the facility versus the laboratory obligation/responsibility in terms of reporting (notifiable medical condition requirements)?**

Since 2017, *C. difficile* became a NMC. All laboratories that identify the *C. difficile* are required to report patients from whom the organism is isolated on a monthly basis to the National Institute for Communicable Diseases.^151^ As the main purpose of the NMC system is to conduct surveillance on a national level, it remains important for healthcare facilities to monitor their own CDI rates for timely monitoring and implementation of control measures.

### H. *Clostridioides difficile* infection prevention and control

#### Summary of recommendations

30. **What infection prevention and control measures should be implemented to minimise the risk of transmission of *Clostridioides difficile*?**

**Recommendation:** Any patient presenting with diarrhoea presents an infectious risk. Early identification is essential and patients considered to have an infectious aetiology (pending investigation – see CDI diagnosis section) should have IPC measures instituted.

Two levels of precautions are recommended for all patients known or presumed to be infected with *C. difficile* (*strong recommendation, moderate quality of evidence*):

- Standard precautions
- Contact transmission-based precautions
  ■ Isolation of patients and dedicated toilet facilities
  ■ Personal protective equipment (PPE): gown/apron and gloves for all patient-care interactions.

31. **Which patients with *Clostridioides difficile* infection should preferentially be isolated?**

**Recommendation:** The following patients should be isolated (*strong recommendation, moderate quality of evidence*):

- Confirmed toxigenic strain of *C. difficile* detected (refer to section on diagnosis) AND incontinence or poor self-hygiene
- Detection of a hypervirulent strain of *C. difficile.*

32. **For how long should isolation precautions remain in place?**

**Recommendation:** Continue contact precautions for at least 48 h after diarrhoea has resolved. Prolong contact precautions until discharge if CDI rates remain high despite control measures being implemented (*weak recommendation, low quality of evidence*).

33. **What are the minimum contact precaution requirements for *Clostridioides difficile* infection patients in a resource-constrained environment (i.e. situation of no single room isolation available)?**

**Recommendation:** When patients cannot be isolated in a single-bed room, a dedicated separate toilet or commode should be allocated when possible. Cohorting of patients with the same organism(s) is recommended if insufficient single rooms for isolation are available (*strong recommendation, low quality of evidence*).

34. **What are the recommendations for hand hygiene in the context of *Clostridioides difficile* infection?**

**Recommendation:** (*strong recommendation, moderate quality of evidence*):

- Gloves should always be worn for all patient-care interactions.
- Wash hands with soap and running water after contact with body substances or any potentially contaminated surfaces (including environment) as well as after removal of gloves and aprons.
- In the absence of running water, alcohol-based hand rub (AHR) should be used.
- Educate patients to wash hands after toilet visits and before eating.

35. **What are the minimum recommendations for environmental cleaning?**

**Recommendation:** (*strong recommendation, low quality of evidence*):

- Daily environmental cleaning and disinfection of rooms of CDI patients, with focus on high-touch surfaces should be done using a sporicidal agent.
- Use disposable equipment where possible or dedicated reusable equipment that it is cleaned and disinfected with a sporicidal disinfectant.
- Following discharge of a patient, rooms/areas should be terminally cleaned and disinfected with a sporicidal agent.
- There are limited data to recommend the use of automated, terminal disinfection after manual cleaning and disinfection; however, consider the use of vaporised hydrogen peroxide and Ultraviolet (UV) light ‘blasting’ if available, as additional disinfection measures after manual cleaning and disinfection, in outbreak and hyper-endemic settings and where there is evidence of repeated cases of CDI in the same isolation area.

##### Rationale for recommendations

30. **What infection prevention and control measures should be implemented to minimise the risk of transmission of *Clostridioides difficile*?**

Two tiers of precautions are recommended for all patients known or presumed to be colonised or infected with *C. difficile*.

Standard precautions are used for the protection of all persons exposed to a hazardous biological agent; they are applied to all patients and in all situations, regardless of diagnosis or presumed infection or colonisation status. Because all patients can serve as reservoirs of infectious agents, adherence to standard precautions during the care of all patients is essential in interrupting the transmission of microorganisms. Detailed information on standard precautions is available in the *Occupational Health and Safety Act, 1993: Regulations for Hazardous Biological Agents.*

The potential transmission role of patients asymptomatically colonised with *C. difficile* has recently been highlighted and illustrates the need for using standard precautions universally.^43^

Standard precautions apply to: (1) blood; (2) all body fluids, secretions and excretions except sweat, regardless of whether they contain visible blood or not; (3) non-intact skin; (4) mucous membranes; and (5) contaminated items, whether or not gloves are worn.

Contact precautions are applied in addition to standard precautions for patients known or presumed to be infected or colonised with epidemiologically important micro-organisms that can be transmitted by direct contact with the patient or indirect contact with environmental surfaces or patient care items in the patient’s environment.

*Clostridioides difficile* transmission occurs most likely from direct contact through person-to-person spread, either from symptomatic patients or from asymptomatic carriers. Another reservoir that facilitates the spread is exposure to a contaminated environment. McDonald et al. suggested that the hands of healthcare workers (HCWs) are probably the main means through which the organism is spread in healthcare settings.^6^ It is thus prudent to identify patients and environments where a high bioburden would contribute to sustained transmission through horizontal transfer. Placing patients on contact precautions theoretically alerts HCWs to a heightened transmission risk and reinforces interventions, such as hand hygiene, to mitigate this risk. However, the evidence supporting this is of low quality and may reflect not only the contribution of other variables to transmission but also the rigour with which contact precautions are implemented and adhered to.^152,153^ A single-centre study has demonstrated that contact precautions are not necessary for all patients and that there is a low risk of transmission (1.3%) of CDI in contact patients exposed for > 24 h through sharing a common room.^152^

Despite the potential role of asymptomatic carriers in the transmission of CDI, neither the IDSA guidelines of 2018 nor the Association for Professionals in Infection Control and Epidemiology (APIC) guidelines of 2013 recommend placing asymptomatic carriers on contact precautions because of insufficient evidence on the efficacy of this measure.

Current guidelines suggest that patients with presumptive CDI should be placed on pre-emptive contact precautions pending test results and that early identification is key; ensure appropriate prompt testing of patients with an acute diarrhoeal illness, not otherwise explained (refer to the CDI diagnosis section).^6,154^

31. **Which patients with *Clostridioides difficile* infection should preferentially be isolated?**

Patients with CDI should ideally be accommodated in a single (private) room with a dedicated or en-suite toilet.^6^ In a resource-constrained setting, this is not always feasible and a decision as to which patients require most isolation may need to be made. In consideration of which patients require isolation, once again one needs to consider which patient poses the greatest transmission risk. In the context of transmission risk, there are potential host and microbe factors to consider. Patients with incontinence or poor hygiene would increase the risk of transmission through extensive environmental contamination. There is also evidence to suggest that patients harbouring a hypervirulent strain are more likely to transmit their strain and cause CDI.^152^ In the absence of isolation facilities, it is recommended to have dedicated toilets for CDI patients.

Cohorting of patients is also an option in a limited resource setting although the evidence for this is of low quality. When cohorting patients, avoid cohorting patients infected or colonised with other multidrug-resistant (MDR) organisms, for example, methicillin-resistant *Staphylococcus aureus* (MRSA) or VRE.^6^

32. **For how long should infection prevention and control precautions be enforced?**

There are varied recommendations on the duration of precautions in preventing CDI. The CDC and the South African Regulations for Hazardous Biological Agents recommend that contact precautions should be maintained for the duration of the illness, whilst others recommend continuing contact precautions for at least 48 h after diarrhoea has resolved.^6,155^

The recommendation from the American Society of Gastroenterology is that contact precautions should be maintained until bowel movements have returned to normal.^156^

In a prospective study of 52 patients, *C. difficile* was suppressed to undetectable levels in stool samples for most patients by the time diarrhoea resolved, with a mean of 4.2 days.^157^ However, at the time of resolution of diarrhoea, skin contamination remained high at 60% and environmental contamination at 37%. Also, *C. difficile* was again detectable in 56% of stool specimens 1–4 weeks after treatment.

There are no studies demonstrating a reduction in CDI incidence by extension of contact precautions. However, as an additional control measure, contact precautions should be extended until discharge if CDI rates remain high despite standard control measures being implemented.

33. **What are the minimum contact precaution requirements in a resource-constrained environment?**

Isolation is a prevention measure used by most healthcare facilities; however, single or private rooms are not always available for infectious CDI patients.

Private rooms facilitate better infection control practices: in a cohort study of healthcare-associated CDI acquisition, higher rates of CDI were demonstrated in patients sharing a double room than in single rooms and there was a significantly higher risk of acquisition after exposure to a roommate with a positive culture result.^158^ Admission to a *C. difficile* cohort ward was shown to be an independent predictor of recurrence of CDI, despite adjustment for potential risk factors, such as age, comorbidities and continued antibiotic use.^159^ It is well recognised that the risk of acquisition of *C. difficile* and other MDR organisms is increased when the previous occupant of a room was colonised with the specific organism in question.^160^ This highlights the potential role of environmental contamination in the transmission and underscores the need for appropriate isolation and environmental decontamination.

Thus, in resource-constrained settings where limited private rooms are available, prioritise patients with stool incontinence for placement in private rooms. If private rooms are not available, cohorting is indicated following the principles outlined above.

34. **What are the recommendations for hand hygiene in the context of *Clostridioides difficile* infection?**

Hand hygiene is considered to be the cornerstone for the prevention of transmission of most contact-driven infections, including CDI. In routine or endemic settings, perform hand hygiene before and after contact with a patient with CDI and after removal of gloves, with soap and water. Handwashing must occur after direct contact with faeces or potentially faecally contaminated areas of the body (e.g. the perineal region).^6^

Transmission of *C. difficile* commonly occurs via the hands of HCWs. When gloves are not worn, 14% – 59% of HCWs’ hands are contaminated after contact with a patient.^6^ Furthermore, studies have demonstrated low rates of handwashing by HCWs, especially when washbasins are not available.^6^ Bacterial spores can be removed from hands by the physical action of washing and rinsing. Hand hygiene compliance has been vastly improved by the introduction of AHRs, which are easy to use at the point of care and which kill most vegetative bacteria and many viruses. However, *C. difficile* spores are highly resistant to inactivation by alcohol and ethanol treatment of stool samples is a recommended step to facilitate the culture of *C. difficile* from stool.^161^ Despite this, studies have not shown an association between the use of AHR and an increased incidence of CDI. It is therefore not conclusive that AHR is inferior to handwashing, despite the lack of *in vitro* activity of ethanol against *C. difficile* spores.^154^

Thus, it is important to confirm and monitor compliance with glove use (including the safe removal of gloves) and with hand hygiene. It is recommended to wash hands with soap and water before and after providing care for CDI patients, rather than using AHR alone although this should not supplant AHR where compliance is high. Although gloving will significantly reduce the degree of contamination of HCWs’ hands, there is still an absolute requirement for optimal hand hygiene after removal of gloves.

Handwashing by patients should be actively encouraged, in particular, after using the toilet and before eating.

35. **What are the minimum recommendations for environmental cleaning?**

*Clostridioides difficile* produces spores that are resistant to drying, heat, detergents and some disinfectants and can therefore survive for months to years in the hospital environment. Affected patients shed spores into their immediate environment, which can serve as the source of transmission to other patients. *Clostridioides difficile* spores have been cultured from commodes, toilets, floors, bed rails, call buttons, sinks and over-bed tables.^6^ It is well documented that environmental contamination occurs as a result of active infection, particularly when patients have large amounts of liquid stool or stool incontinence. Environmental contamination has been found to be the highest in rooms of patients with CDI, lower in rooms of asymptomatic carriers and the lowest in rooms of culture-negative patients.^6^ Heavy contamination occurs on floors, commodes, toilets, bedpans and bed frames.^155^

Single-use disposable equipment should be used to prevent CDI transmission wherever available. Any re-usable equipment should be dedicated to the isolation area until the patient has been discharged and/or it has been thoroughly cleaned and effectively disinfected. It is recommended that the cleaning and disinfection methodology and responsibility for decontamination are clearly addressed in standard operating procedures. *Clostridioides difficile* transmission has been associated with contaminated commodes, blood pressure cuffs and oral and rectal electronic thermometers.^6^

Cleaning with detergents may be insufficient for environments contaminated with *C. difficile* and sub-inhibitory concentrations of disinfectants may enhance sporulation.^155^ Daily sporicidal disinfection has been associated with reductions of CDI in outbreak settings (in conjunction with other measures). Mayfield et al. demonstrated that a hypochlorite-based solution at 5000 parts per million (ppm) available chlorine (0.5% solution) reduced the incidence of CDI in a bone marrow transplant unit, where the baseline incidence of CDI was relatively high. When the original quaternary ammonium compound was re-introduced, CDI increased to almost the baseline level.^162^ A study by Orenstein et al. found that daily use of bleach wipes containing 0.55% active chlorine decreased CDI by 85% in two units with hyperendemic rates and another study showed a reduction in contamination of HCW’s hands when high-touch surfaces were disinfected daily with a peracetic acid-based disinfectant.^163,164^ However, no studies have compared daily cleaning against terminal cleaning only with a sporicidal agent, which has also been shown to decrease the incidence of CDI.^165^

Reductions in viable *C. difficile* spores in the environment have been associated with improved compliance with thoroughness of cleaning by a trained, dedicated team of environmental cleaners. Barriers to effective cleaning included insufficient time, inadequate cleaning supplies, inadequate education and poor communication.

Terminal disinfection with a sporicidal agent (in conjunction with other measures to prevent CDI) has been associated with reductions in CDI in outbreak settings. This has not consistently been shown for CDI in non-outbreak, endemic settings, where the turnover of CDI patients in a room is lower and the contamination of the room is not sufficient to cause transmission, especially when there is daily cleaning, which removes *C. difficile* spores. Other confounding variables in studies include the use of different disinfection products (sodium hypochlorite, phenols, peroxides and UV irradiation), applied manually or by automated systems, with different regimens of cleaning: daily cleaning alone, daily and terminal cleaning, terminal cleaning alone and ‘deep cleaning’.

A modelling study evaluating environmental strategies aimed at different agents of *C. difficile* transmission within a hospital environment identified daily sporicidal cleaning and screening for asymptomatic carriage as the two most effective single interventions for reducing rates of hospital-onset CDI.^166^ The strength of this study was that different transmission events were simulated to account for the complexity of hospital IPC dynamics, and to highlight the potential role of future mathematical modelling in IPC interventional studies. The study evaluated nine different IPC interventions and the results suggested that unidentified environmental sources of *C. difficile* should be the primary target for intervention.

Automated disinfection technologies such as ultraviolet radiation (UVR) and hydrogen peroxide vapour (HPV) have been found to reduce viable *C. difficile* spores in patient rooms.^167^ According to the IDSA guidelines, most studies have at least one significant limitation precluding any definitive statement on the use of automated disinfection technology as a core component of a CDI prevention programme.^6^ Moreover, further data subsequent to the 2018 IDSA guidelines including the Benefits of Enhanced Terminal Room (BETR) Disinfection study did not demonstrate any reduction in *C. difficile* acquisition and infection by the addition of UVR compared to terminal disinfection with bleach.^168^ In a secondary analysis of this data set, which assessed the hospital-wide, hospital-acquired incidence of CDI, addition of UVR to standard disinfection reduced the incidence of CDI. It is unclear however why this benefit was not seen in the bleach and UVR study period, although it suggests some indirect effect of UVR on the acquisition of *C. difficile* when targeted high-risk rooms are exposed to UVR.

### I. Managing *Clostridioides difficile* infection in an outbreak setting

#### Summary of recommendations

36. **What additional infection prevention and control measures should be applied in an outbreak setting?**

**Recommendation 1:** Surveillance (*strong recommendation, low quality of evidence*)

- Define the threshold and monitor incidence and testing rates (as per surveillance recommendations) with timely feedback.
- Inform all HCWs about the outbreak and conduct a risk assessment:
  ■ Determine location of new CDI cases.
  ■ Review unnecessary use of antimicrobial agents (inform antimicrobial stewardship team [AMS] team if applicable).
  ■ Assess and monitor compliance with instituted IPC interventions.

**Recommendation 2:** Contact precautions and personal protective equipment

- Contact precautions including isolation and PPE (gloves and disposable gowns/aprons) for all patients with new-onset diarrhoea (*strong recommendation, low quality of evidence*).

**Recommendation 3:** Hand hygiene

- Monitor and target improvements in hand hygiene compliance (*strong recommendation, low quality of evidence*).
- Ensure hand washing with soap and water in preference to AHR (*conditional recommendation, low quality of evidence*).

**Recommendation 4:** Environmental cleaning and disinfection

- Reinforce cleaning and hygiene measures (*strong recommendation, low quality of evidence*).
- Ensure daily and regular cleaning of the unit with a sporicidal agent after initial cleaning with a detergent, for example, hypochlorite 1:1000 ppm (*strong recommendation, low quality of evidence*).
- Terminal cleaning with sporicidal agent after initial cleaning, for example, hypochlorite 1:1000 ppm (*strong recommendation, low quality of evidence*).
- There are limited data to recommend the use of vaporised hydrogen peroxide and UV light disinfection (*no recommendation, low quality of evidence*).

**Recommendation 5:** Screening

- Identification of asymptomatic carriers through routine screening is not recommended (*conditional recommendation, low quality of evidence*).
- Screening of HCW for *C. difficile* colonisation is not recommended (*strong recommendation, low quality of evidence*).

##### Rationale for recommendations

36. **What additional infection prevention and control measures should be applied in an outbreak setting?**

An outbreak is defined as an increase in the number of cases above a certain threshold of what is expected. The identification, subsequent investigation and then implementation of interventional measures require surveillance data. A risk assessment and analysis of potential contributory factors should be undertaken using surveillance data. Given the influence of diagnostics on defining a case of CDI, it is important to establish clinically whether cases truly represent CDI versus colonisation as the outbreak may be spurious, an artefact of increased testing and detection especially where highly sensitive NAAT are used as a sole means to diagnose CDI. In a suspected outbreak situation, it is recommended to ascertain and stratify all cases in terms of true hospital-onset, healthcare facility-associated community onset and community onset. Furthermore, additional surveillance rates stratification by location is recommended. Communication and relaying of information is crucial to all relevant stakeholders.

The identification of an outbreak should prompt an evaluation of all relevant IPC measures aimed at reducing rates of CDI. It is imperative that all IPC measures are reinforced and any breaches are rectified as soon as possible. It is often possible to halt an outbreak through prompt identification of lapses in IPC measures, and subsequent reinforcement of these measures. Reported studies addressing CDI outbreaks typically employ a ‘bundle’ approach to contain the outbreak. Because of the retrospective observational design of these studies, it is difficult to ascertain if any single intervention carries more weight and should be preferentially implemented over others. Furthermore, there are no studies from Africa or South Africa that take into account the unique challenges and varied hospital/healthcare facility settings, which are prevalent throughout the continent. Based on this, all the provided recommendations have a low to very low quality of evidence base, yet the strength of the recommendation is supported by these interventions having either established benefit in controlling CDI in endemic settings or a good clinical practice basis.^154^

### J. Antimicrobial stewardship and *Clostridioides difficile* infection

#### Summary of recommendations

37. **Should *Clostridioides difficile* infection rates be included in antimicrobial stewardship programmes (ASP) as an outcome measure?**

**Recommendation:** It is recommended to use CDI rates as a tool to monitor the effectiveness of an ASP, on condition that the IPC programme can support this metric (*conditional recommendation, low quality of evidence*).

38. **What antimicrobial stewardship measures should be instituted to reduce rates of *Clostridioides difficile* infection?**

**Recommendation:**

- Restriction of certain agents/classes is recommended to reduce rates of CDI (*strong recommendation, low-to-moderate quality of evidence*)

and

- Reducing the duration of antimicrobial therapy is recommended to reduce rates of CDI (*strong recommendation, low quality of evidence*).

##### Rationale for recommendations

37. **Should *Clostridioides difficile* infection rates be included in antimicrobial stewardship programmes (ASP) as an outcome measure?**

The rationale for including CDI rates in ASP’s metrics relates to the following:

A recent systematic review and meta-analysis found that ASPs effectively reduced the incidence of both infection and colonisation with multidrug-resistant (MDR) Gram-negative bacteria, MRSA and CDI.^169^

In-hospital CDI rates may also be used in goal setting as national targets for reducing antibiotic-resistant infections by a certain time – in the case of the USA, a 50% reduction by 2020.^170^

Antibiotic misuse and overuse facilitate the development of multidrug-resistant organisms (MDROs), as well as CDI infections – an antibiotic-associated adverse drug event – making antimicrobial stewardship an important synergistic HAI prevention and control strategy.^169,171^

Benchmarking rates of CDI across multiple hospitals, identification of the causes of inter-hospital variability in rates and the best strategies to reduce rates seem to be important areas of outcomes research in stewardship.^172^

Intuitively, the strong association between antibiotic use and CDI makes it appealing to use CDI rates as a measure of the effectiveness of antimicrobial stewardship interventions. However, as outlined in the aforementioned sections of this guidance document, there are a number of potential pitfalls that must be considered prior to utilising CDI rates as a measure of ASP. These relate not specifically to the ASP intervention(s) itself but rather to the surveillance and diagnosis aspects of CDI. These two aspects of CDI are highly variable and not currently standardised, although the purpose of this document is primarily to provide guidance on these issues, which may facilitate standardisation.

In the context of surveillance, it is crucial to understand the burden of CDI within a facility, both in terms of incident cases (hospital-acquired) and admission prevalence (community-acquired or healthcare facility-associated community onset). Without distinguishing these cases at a facility level, the impact of any ASP interventions will be unclear. Thus, the use of CDI rates as a tool to monitor effectiveness of an ASP requires a robust surveillance system that is able to accurately categorize CDI in terms of facility/ward incidence and prevalence as outlined in the current CDC MDRO protocol.^147^ This degree of surveillance is beyond that which is recommended in this guidance document although the recommendations made here are designated the minimum requirement and facilities which have capacity to perform enhanced surveillance should aim to do so.

38. **What antimicrobial stewardship measures should be instituted to reduce rates of *Clostridioides difficile* infection?**

In the context of diagnosis, detection of toxigenic strains by highly sensitive NAAT assays does not necessarily imply active CDI with disease. The asymptomatic colonised patients or patients with diarrhoeal illness attributable to other causes who test positive for a toxigenic strain are currently included in the calculation of CDI rates. Again, the inclusion of these cases may mask any ASP interventions and the impact of CDI rates. On this basis, a conditional recommendation for inclusion of CDI rates as an ASP measurement tool is made, the caveat being that surveillance of CDI for calculation of rates must take into account both the diagnostic elements and the detailed categorisation of CDI events.

Judicious use of antimicrobial agents is a key principle of AMS and thus any avoidance of unnecessary use of antimicrobials and reducing the duration of use should be strongly and actively encouraged. *Clostridioides difficile* infection is an unintended consequence of antimicrobial use and, thus, limiting frequency and duration of antimicrobial use will reduce CDI rates.^6^ Although virtually all antimicrobials have been linked to CDI, certain classes are more commonly linked and have a higher propensity for the development of CDI. These include extended-spectrum cephalosporins, clindamycin and fluoroquinolones. The widespread empiric use of these agents should be avoided and they should be reserved for targeted applications. The use of multiple antimicrobials has also been shown to be an important risk factor for the development of CDI and thus it is important to reduce cumulative exposure.^6^ It is important to note that ASP-targeting antimicrobial use has been shown to have a greater impact on CDI rates when co-implemented with IPC measures, especially hand hygiene.^171^ In this context, an ASP should be seen as part of the greater IPC programme and not as a stand-alone programme. The greatest benefit in reduction of CDI rates will be realised through interventions that capture the synergistic effects of antimicrobial stewardship and IPC.

### K. *Clostridioides difficile* infection prevention

#### Summary of recommendations

39. **Should proton pump inhibitor use be restricted to reduce *Clostridioides difficile* infection incidence?**

**Recommendation:** Inadequate evidence to make recommendation.

40. **What is the role of probiotics in the prevention of *Clostridioides difficile* infection?**

**Recommendation**: Inadequate evidence to make recommendation.

##### Rationale for recommendations

39. **Should proton pump inhibitor use be restricted to reduce *Clostridioides difficile* infection incidence?**

Proton pump inhibitors (PPI) are commonly prescribed drugs. However, between 25% and 70% of these prescriptions are inappropriate.^173^ Meta-analyses have shown an association between PPI use and the risk of developing CDI.^174,175^ These analyses, however, reported substantial clinical and statistical heterogeneity. Contributing factors may be the inclusion of studies with non-PPI acid-suppressing drugs, different definitions of CDI, community- and hospital-acquired cases, varying hospital departments, and initial and recurrent CDI.^176^ Proton pump inhibitors can also cause diarrhoea, resulting in more frequent investigation of CDI in comparison to that in patients who are not receiving PPIs.^24^ Confounding because of greater severity of illness in CDI patients (with more ill patients being more likely to be prescribed PPIs) compared to selected controls is an additional factor that needs consideration.^24^

A cumulative meta-analysis that included 50 studies from 2000 to 2016 found that since 2011 the association between PPI use and CDI risk has been relatively constant, with an OR of 1.20–1.26.^174^

A lack of RCTs or other appropriate evidence to show the causality remains, preventing the formulation of clear recommendations regarding the restriction of PPIs for CDI prevention when the PPI use is otherwise indicated. However, inappropriate PPI use must be stopped.

40. **What is the role of probiotics in the prevention of *Clostridioides difficile* infection?**

A Cochrane review undertaken in 2017 found that in populations with a > 5% baseline risk of CDI, probiotics were associated with a 70% reduction in CDI risk (*p* = 0.01; moderate quality of evidence).^177^ In lower clinical risk scenarios, probiotics showed no benefit. A CDI incidence of > 5% is unusual even in an outbreak setting.^178^ Limitations of this review: trials from differing clinical settings (inpatients and outpatients), and varying probiotic formulations and doses were analysed together.^179^ In addition, the trials in the review did not include immunocompromised patients. No severe adverse events (SAE) were reported in the included trials. The authors concluded that probiotics seem to be safer in non-immunocompromised and non-severely debilitated patients.

An individual patient data meta-analysis similarly reported that probiotics are useful for CDI prevention in hospitalised patients, with a ≥ 5% risk for CDI and a lack of SAE.^180^

The administration of probiotics can result in infection.^181^ Further research is needed to establish which patient populations will benefit most from probiotic prophylaxis and the risk–benefit ratio in immunocompromised patients.^179^

## Future directions

Ongoing research and development is required to improve the diagnosis, treatment and prevention of CDI. Current research and areas requiring further investigation, particularly for the South African context, are outlined.

### Diagnosis

1. Validated multivariable prediction rules to determine the pretest probability of CDI, to guide the testing strategy.
2. Local well-designed prospective diagnostic accuracy studies that include pre-analytic factors such as consistency of stool samples tested and pretest probability of CDI, as well as post-analytic clinical outcomes, particularly for algorithm-based testing.
3. Ultrasensitive toxin detection assays may improve the sensitivity of *C. difficile* toxin detection in clinical laboratories and may result in more widespread use of toxin detection assays (rather than only NAAT-based testing) in South Africa.^182^ The Singulex Clarity *C. difficile* toxin A/B assay has a limit of detection in stool for toxins A and B of 2.0 pg/mL and 0.7 pg/mL, respectively. Laboratory comparisons have shown the assay to have an equivalent sensitivity to PCR and 100% specificity.^183^ Assays such as this have the potential to be used as stand-alone diagnostic tests and possibly reduce unwarranted treatment.

### Treatment

1. Good clinical trials for the management of rCDI. The OpTION study (NCT02667418) is designed to provide much needed data on the comparative efficacy of standard fidaxomicin therapy and standard vancomycin therapy, followed by a tapered and pulsed course.
2. Establishment of registries in South Africa for FMT recipients is important. Monitoring for unintended long-term sequelae is indicated.
3. Determining the role of bezlotoxumab in various patient populations. MODIFY III (NCT03182907) is looking at pharmacokinetics, safety and tolerability of bezlotoxumab in children with CDI. ICON-2 (NCT03829475) is comparing the effects of FMT plus bezlotoxumab with FMT plus placebo in patients with both IBD and CDI. When bezlotoxumab is approved for use in South Africa, evidence supporting its use in specific patient populations will determine its role in rCDI prevention strategies.

### Prevention

1. The role of oral vancomycin or fidaxomicin for the prevention of rCDI when systemic antibiotics for the treatment of other infections are required following an episode of CDI. Clarity is required regarding which, if any, patient populations are likely to benefit from prophylaxis (e.g. patients receiving antibiotics associated with a high risk for CDI, patients with single or multiple prior CDI episodes and time elapsed from last CDI episode), dosing regimen for oral vancomycin, the associated risk of VRE infections and the possible deleterious impact on the gut microbiome.
2. Additional assessment and study on the impact and cost-effectiveness of various IPC interventions at a local level is required. As our understanding of *C. difficile* colonisation and transmission dynamics develops, it is evident that many traditional practices may not be relevant in controlling or preventing the spread of *C. difficile.* This is particularly important in a resource-constrained environment and adoption of practices needs to take into account both the evidence base and the prevailing healthcare environment or practice.
3. From an African perspective, the clinical and molecular epidemiology of the disease is poorly understood and future initiatives should focus on enhancement of surveillance. As outlined in this guidance document, standardised reporting of rates by facilities is imperative; however, additional epidemiological data are essential and this may require the establishment of a centralised facility that can coordinate enhanced surveillance and provide centralised testing (including molecular analysis) of submitted isolates.
